# Impact of metallurgical and geometric characteristics on cyclic fatigue resistance of three thermally treated NiTi rotary instruments

**DOI:** 10.4317/jced.63479

**Published:** 2026-03-30

**Authors:** Raimundo Sales de Oliveira Neto, Guilherme Ferreira da Silva, Diego Rafael Nespeque Correa, Rodrigo Ricci Vivan, Murilo Priori Alcalde, Marco Antonio Hungaro Duarte

**Affiliations:** 1 Department of Operative Dentistry, Endodontics and Dental Materials, Bauru School of Dentistry, University of São Paulo – USP, Bauru, Brazil; 2 São Paulo State University (UNESP), School of Sciences, Campus Bauru, Laboratory of Anelasticity and Biomaterials, Bauru, Brazil

## Abstract

**Background:**

This study aimed to evaluate the cyclic fatigue resistance and metallurgical characteristics of thermally treated NiTi rotary instruments: ProDesign Logic, Solla Purple, and Solla Colors.

**Material and Methods:**

Cyclic fatigue testing was conducted at room temperature (20°C ± 1°C) and simulated body temperature (36°C ± 1°C) using a standardized artificial canal with a 60° curvature and 5 mm radius. Ten instruments per group (25.06) were tested at each temperature. Differential Scanning Calorimetry (DSC) and Scanning Electron Microscopy (SEM) were employed to assess phase transformation behavior and fracture topography, respectively.

**Results:**

ProDesign Logic exhibited the highest cyclic fatigue resistance at room temperature compared to Solla systems (p&lt;0.0001). Temperature variations did not significantly affect cyclic fatigue resistance (p&gt;0.05). ProDesign Logic had the highest austenite finish temperature (Af = 53.0°C). SEM confirmed typical fatigue fracture patterns in all groups.

**Conclusions:**

ProDesign Logic demonstrated greater cyclic fatigue resistance, attributed to its high Af temperature and cross-sectional design.

## Introduction

Nickel-titanium (NiTi) endodontic instruments are widely used in root canal preparation due to their flexibility and ability to shape curved canals. However, instrument separation can occur during canal instrumentation, with cyclic fatigue being a primary cause of fracture (1 , 2). Cyclic fatigue arises when a NiTi instrument rotates within a curved canal, subjecting it to repeated cycles of tension and compression. The constant stress concentration at the point of maximum curvature leads to progressive material fatigue, ultimately resulting in instrument fracture (3 - 5). To enhance the fracture resistance of NiTi files, manufacturers have developed new instruments through design modifications, surface and thermal treatments (6). These thermal treatments have enabled the production of controlled-memory (CM) instruments, which primarily exhibit a martensitic crystalline structure at room temperature. This characteristic allows the instrument to return to its original shape upon heating, because of the phase transition (martensitic crystallographic phase to austenitic phase) (7 , 8). Compared to conventional/superelastic instruments, CM files demonstrate superior resistance to both cyclic and torsional fatigue (9 , 10). In addition to mechanical testing (e.g., cyclic and torsional fatigue assessments), metallurgical characterization enables a more comprehensive evaluation and a deeper understanding of the mechanical properties of new instruments. This approach provides a physicochemical explanation for their mechanical behavior (1 , 11 , 12). According to a previously published literature review, differential scanning calorimetry (DSC) is the most frequently reported metallurgical analysis in the literature (13). DSC is primarily used to determine enthalpy changes and phase transformation temperatures of NiTi alloys. The combination of DSC analysis and mechanical testing enables a more accurate and clinically relevant assessment of the properties of new NiTi instruments (11). The ProDesign Logic instruments (Easy Bassi, Belo Horizonte, Minas Gerais, Brazil) are manufactured from thermally treated CM NiTi. These instruments are available with various tip sizes and tapers, featuring distinct cross-sectional designs: a double-helix configuration for instruments with .03, .05, and .06 tapers; a triple-helix design for .04 taper instruments; and a quadruple-helix geometry for .01 taper instruments. Previous studies have demonstrated that these instruments exhibit superior cyclic and torsional fatigue resistance compared to other commercially available systems (3 , 14 , 15). Although their mechanical properties have been extensively evaluated, no studies to date have investigated the metallurgical characteristics of the ProDesign Logic system. Recently, two new NiTi instrument systems have been introduced to the market: Solla Purple and Solla Colors (Shenzhen Forever Medical, Longgang District, Shenzhen, China). The rotary Solla Purple system features a triangular cross-section (inactive tip) for its smaller instruments (#15.04, #20.05, and #25.06) and a trapezoidal cross-section for larger files (#35.04). The Solla Purple system undergoes a proprietary 'Purple' manufacturing process, while the Solla Colors instruments feature a 'Colors' treatment. However, the manufacturer's specifications do not clearly distinguish whether these distinctive colorations result from thermal or surface treatments. Despite their commercial availability, no studies have yet evaluated their metallurgical properties and mechanical performance, highlighting a significant gap in the current literature. Given the critical importance of mechanical and metallurgical properties in ensuring clinical safety and case-specific instrument selection, this study aims to comprehensively evaluate the performance characteristics of ProDesign Logic, and the newly introduced and commercially available Solla Colors and Solla Purple files.

## Materials and Methods

**Figure 1:**
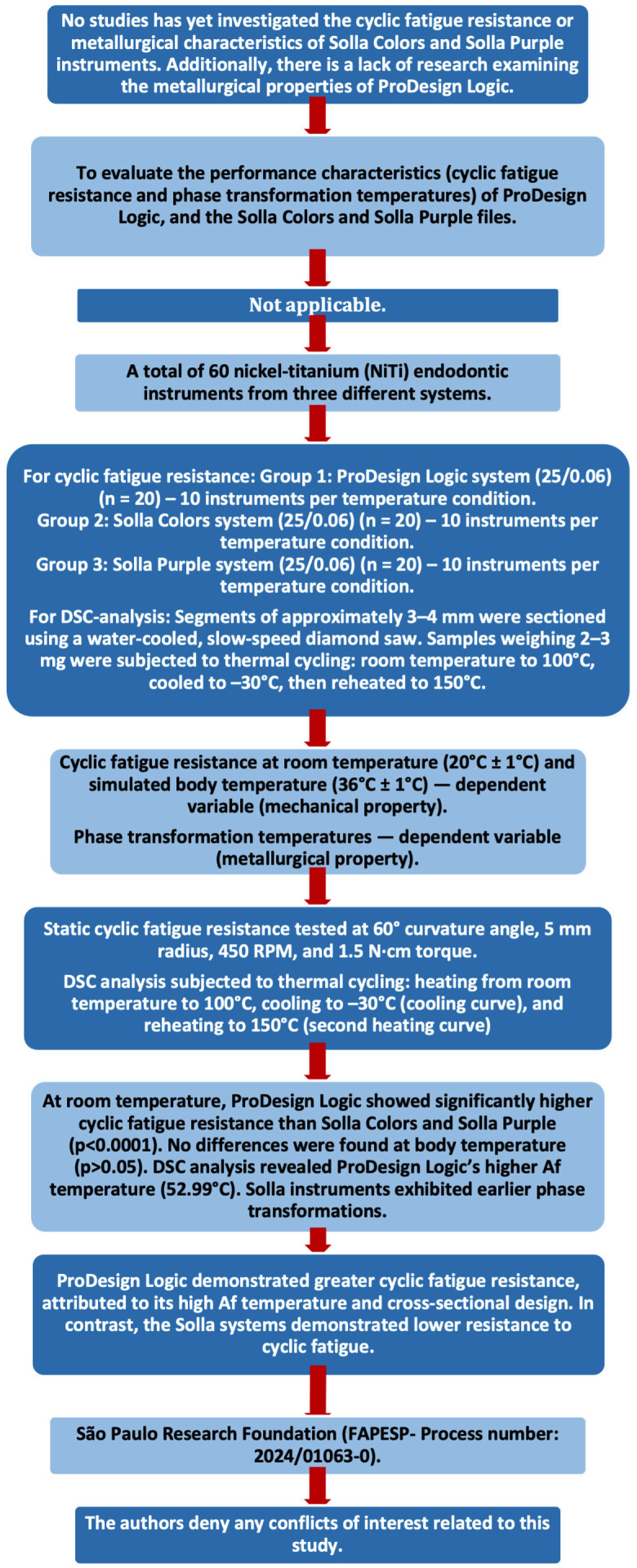
PRILE Flowchart.

The manuscript of this laboratory study has been written according to Preferred Reporting Items for Laboratory studies in Endodontology (PRILE) 2021 guidelines (Fig. 1).

1

- Sample size calculation The sample size was determined through power analysis using G*Power 3.1 (Heinrich Heine University, Düsseldorf, Germany), which indicated that 20 instruments per group (10 per temperature condition) were needed to reliably detect differences in cyclic fatigue resistance, based on an effect size of 1.8 from previous data (5) with 95% power and = 0.05. - Cyclic fatigue Cyclic fatigue testing was conducted at room temperature (20°C ± 1°C) and simulated body temperature (36°C ± 1°C). For maintaining precise temperature control, a histology water bath (Leica HI 1210; Leica Biosystems, Nussloch, Germany) was used to regulate body temperature. The container was filled with 600 mL of water to ensure complete submersion of the simulated canal. Temperature was monitored throughout the test using both the water bath's digital thermometer and an external infrared thermometer (5 , 11 , 16). Six instruments of each type (n = 6 per group) were tested at each temperature, including Solla Purple #25.06, Solla Colors #25.06, and ProDesign Logic #25.06. Before testing, all instruments were examined for defects or deformities under a stereomicroscope (Stemi 2000C; Carl Zeiss, Jena, Germany) at 16× magnification to ensure structural integrity. The cyclic fatigue test was conducted using an apparatus designed to simulate the curvature of an artificial stainless-steel canal, as detailed in prior studies (3 - 5 , 17). This apparatus featured a 60° curvature and a 5 mm radius. The curvature adjustment was achieved using a guide cylinder (5 mm radius) and an outer arc with a 1 mm deep groove, facilitating the rotation of instruments while maintaining them within the curvature. An electric motor, VDW Reciproc Silver (VDW, Munich, Germany), was coupled to this apparatus, and instruments were operated with 450 RPM and 1.5 N.cm of torque. The time taken for instrument fracture was measured using a digital stopwatch and corroborated by simultaneous filming. - Differential Scanning Calorimetry (DSC) analysis Differential scanning calorimetry (DSC) analysis (Mettler Toledo, Barueri, São Paulo, Brazil) was performed to determine the phase transformation temperatures of the NiTi alloy, by ASTM standards (18). The austenite start (As) and austenite finish (Af) temperatures were assessed, as these transitions are clinically relevant when instruments move from a cooler environment to the warmer root canal, mirroring the thermal process simulated by DSC (19). For analysis, segments approximately 3-4 mm in length were sectioned using a water-cooled, slow-speed diamond saw. Samples weighing 2-3 mg were placed in open aluminum pans and subjected to the following thermal cycle: heating from room temperature to 100°C, cooling to -30°C (to record the cooling curve) and reheating to 150°C (to obtain the second heating curve). - Scanning Electron Microscopy (SEM) analysis Transverse images of the fractured instruments were obtained using a scanning electron microscope (SEM - Aspex Express Fei Europe, Eindhoven, the Netherlands) to determine the topographic features of the fractured surface. Before the SEM evaluation, the instruments were ultrasonically cleaned (L100, Schuster, Santa Maria, RS, Brazil) in a saline solution for 3 minutes. The instruments were examined at ×200 magnification. - Statical Analysis Normality of the data was verified using the Shapiro-Wilk test. For intergroup comparisons under the same temperature condition, one-way ANOVA followed by Tukey's post hoc test was applied. Pairwise comparisons between instruments tested at room temperature and simulated body temperature were performed using unpaired two-tailed Student's t-tests. A significance level of 5% was adopted for all analyses. Statistical procedures were conducted using GraphPad Prism 9.3 (GraphPad Software Inc., San Diego, CA, USA).

## Results

Table 1 presents the times in seconds and the number of cycles to failure (NCF) of the examined instruments.

Table 1

**Figure 2:**
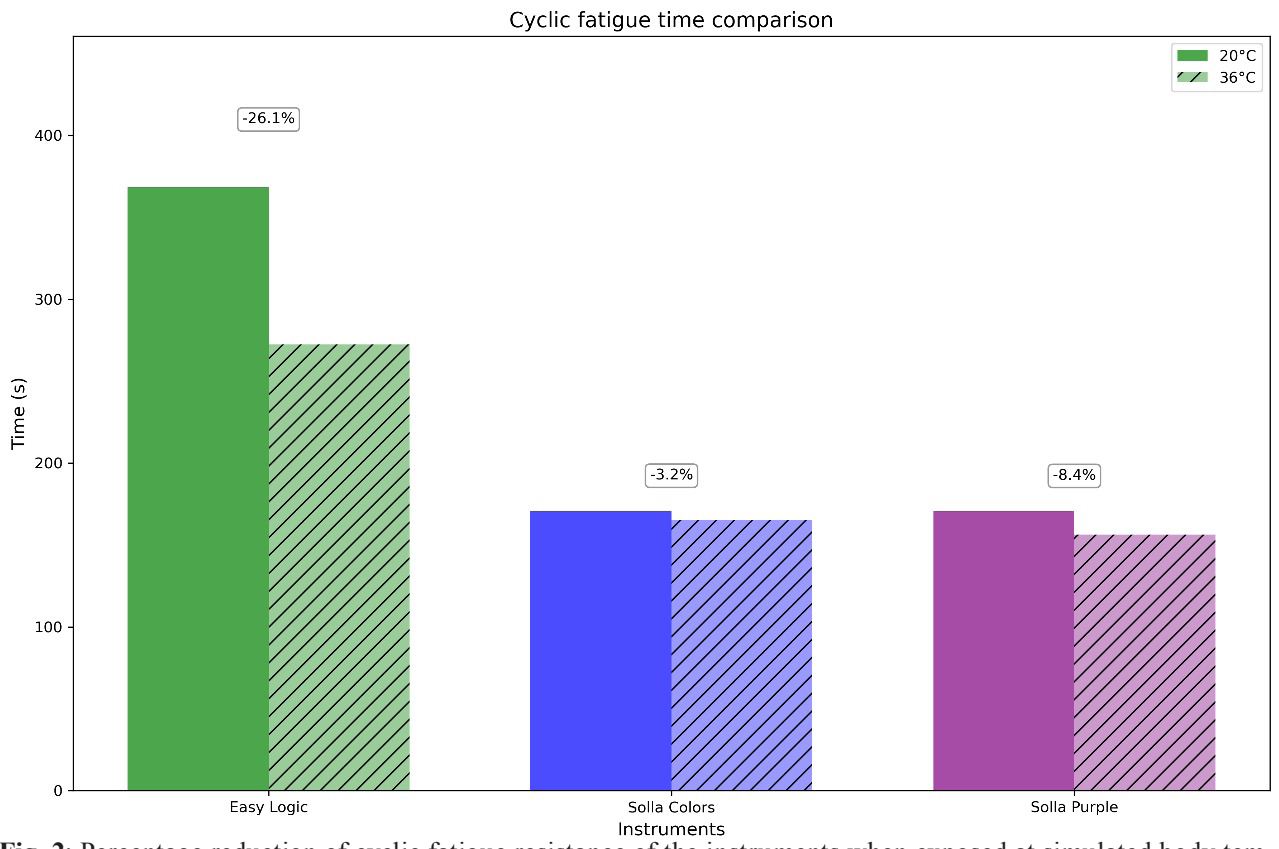
Percentage reduction of cyclic fatigue resistance of the instruments when exposed at simulated body temperature (36°C ± 1°C).

In addition, the percentage of reduction in cyclic fatigue at body temperature was calculated, and these data are also shown in Figure 2.

2

**Figure 3:**
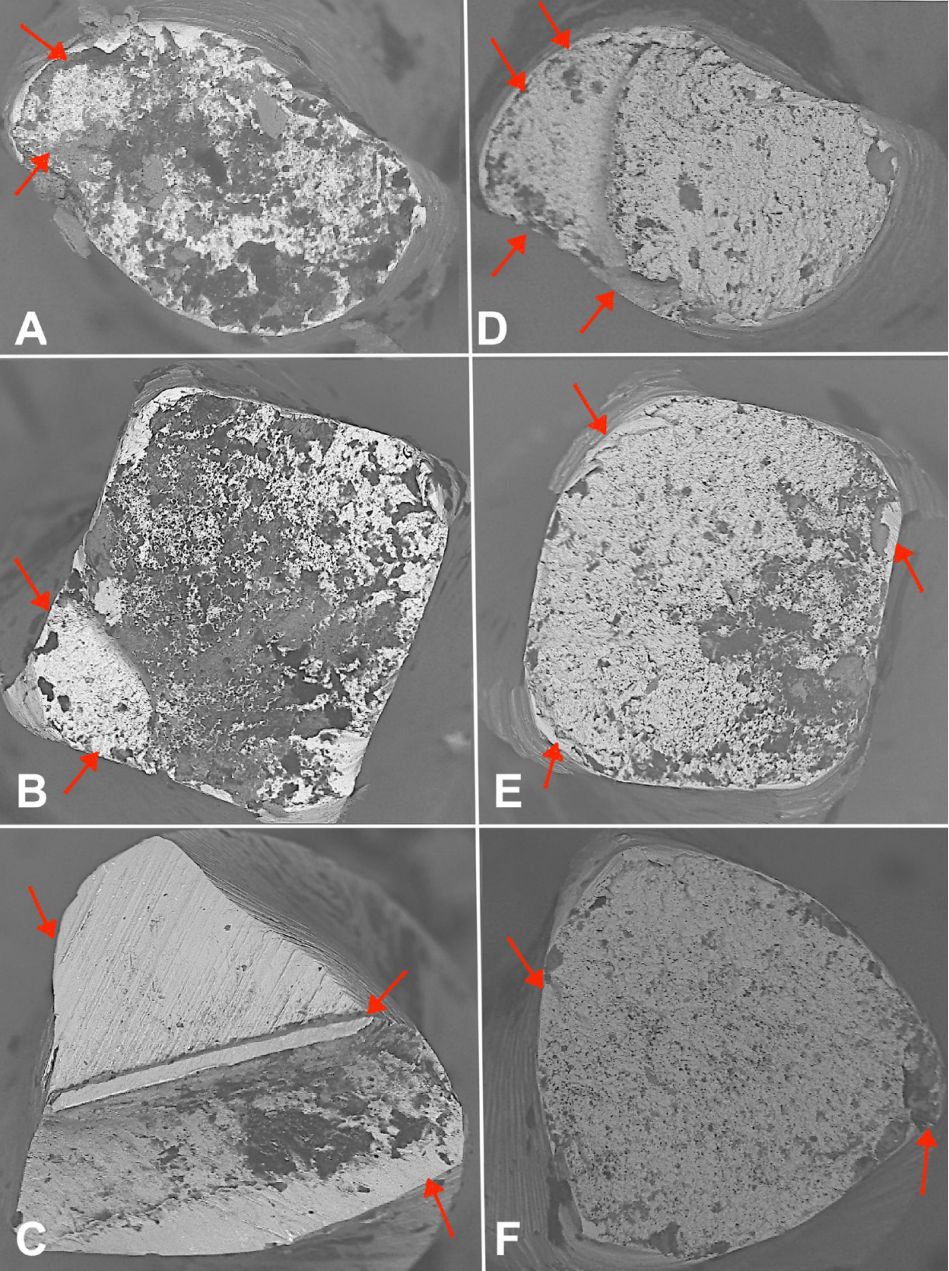
Scanning electron microscopy (SEM) images (200× magnification) of fractured surfaces after cyclic fatigue testing. (A) ProDesign Logic at room temperature (20°C), (B) ProDesign Logic at simulated body temperature (36°C), (C) Solla Colors at room temperature, (D) Solla Colors at simulated body temperature, (E) Solla Purple at room temperature, and (F) Solla Purple at simulated body temperature. Red arrows identify the crack origins. All images exhibit characteristic fatigue striations and microstructural features associated with cyclic failure.

At room temperature, ProDesign Logic exhibited significantly higher cyclic fatigue resistance (time and NCF) compared to Solla Purple and Solla Colors (p&lt;0.0001), with no difference between the Solla instruments (p&gt;0.05). At body temperature, no significant differences were observed among instruments (p&gt;0.05). Temperature did not significantly alter the performance of any individual instrument (all p&gt;0.05). Figure 3 displays SEM images of the fractured instruments from each system.

3

**Figure 4:**
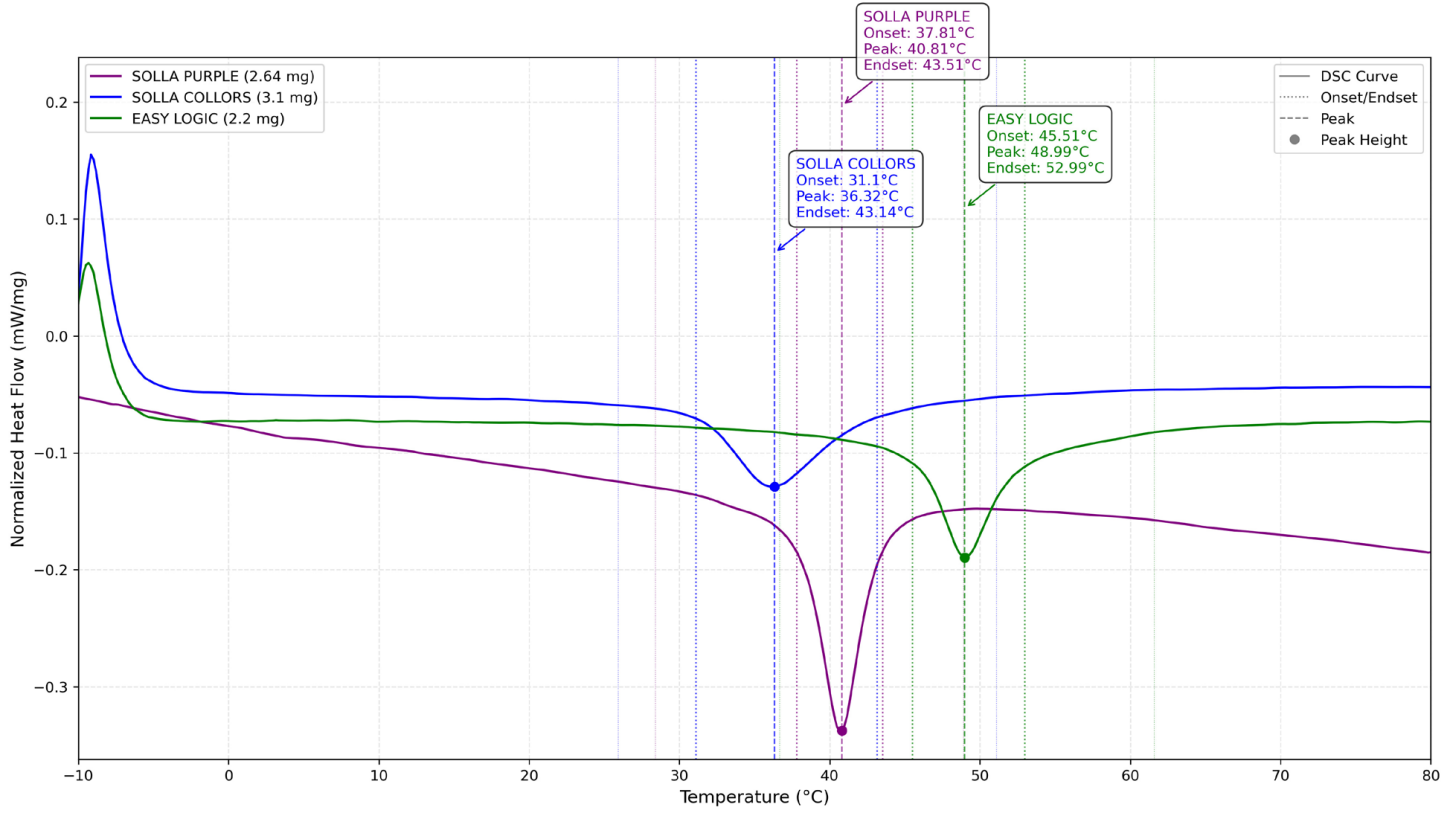
Differential scanning calorimetry (DSC) heating curves of the tested endodontic instruments (read from left to right). The blue curve represents Solla Colors, the purple curve represents Solla Purple, and the green curve represents ProDesign Logic. Critical phase transformation temperatures are annotated: onset (As, austenite start), peak (Ap, austenite peak), and endset (Af, austenite finish).

These images depict characteristic features indicative of cyclic fatigue (crack initiation zones with fatigue striations, progressive crack propagation areas showing plastic deformation of lateral cutting edges, and overload zones containing numerous microvoid coalescence dimples) behavior observed under SEM. The DSC analysis of the second heating cycle revealed distinct phase transformation characteristics among the endodontic instruments. Solla Purple exhibited an Af temperature of 43.5°C, indicating a mixed martensite-austenite phase at body temperature (37°C) with moderate superelasticity (H = -5.06 J/g), consistent with conventional NiTi or M-Wire alloys. Solla Collors showed a low transformation start temperature (As = 31.1°C) and broader peak width (6.79°C), suggesting the presence of R-phase and microstructural heterogeneity characteristic of CM-Wire alloys. ProDesign Logic demonstrated the highest Af temperature (53.0°C), maintaining pure martensite at 37°C with, a characteristic typical of high-Af thermally treated alloys, such as CM-Wire. All data derived from the second heating cycle DSC (ASTM F2004-17), representing stabilized material behavior post-thermal cycling. Figure 4 presents the DSC curves comparing the second heating cycle of Solla Purple, Solla Colors, and ProDesign Logic endodontic instruments.

4

The normalized heat flow (mW/mg) is plotted against temperature (°C), showing distinct phase transformation behaviors from martensite to austenite. Key transformation temperatures (onset, peak, and endset) are annotated for each instrument.

## Discussion

While endodontic NiTi file systems are continuously developed and marketed to enhance clinical efficiency and procedural safety, many newly introduced systems, including the Solla files (Purple and Colors), enter clinical use without a comprehensive evaluation of their mechanical and metallurgical properties. This study provides a critical characterization of these instruments by systematically assessing both their phase transformation behavior and cyclic fatigue resistance. Also, this study provides the first metallurgical characterization of ProDesign Logic instruments. The DSC results provide a metallurgical basis for the observed cyclic fatigue performance (11 , 20). ProDesign Logic's superior fatigue resistance at room temperature aligns with its high austenite finish temperature (Af = 52.99°C), which enables it to maintain a stable martensitic structure during instrumentation. This phase stability, coupled with narrow thermal hysteresis (6.3°C), minimizes energy dissipation during cyclic loading, delaying crack initiation (21). In contrast, Solla Colors and Solla Purple exhibited lower NCF values, consistent with their earlier phase transformations (Af = 43.14°C and 43.5°C). While Solla Colors' R-phase presence (evidenced by low As = 31.1°C and broad DSC peaks) can enhance stress distribution, its heterogeneous microstructure likely introduced localized stress concentrators (8 , 22). Solla Purple's conventional NiTi behavior (H = -5.06 J/g) and mixed-phase composition at 37°C further limited its cyclic fatigue resistance, as the absence of advanced thermal treatments may reduce energy absorption capacity during cyclic deformation (6 , 14 , 17 , 19). Temperature variations did not significantly alter the cyclic fatigue resistance of the tested #25.06 instruments (p&gt;0.05), despite DSC-predicted phase differences. These findings suggest that instrument-specific design characteristics - particularly the different cross-section designs of ProDesign Logic (modified S-shaped), Solla Purple (triangular), and Solla Colors (trapezoidal) - combined with their distinct thermal treatments may have a greater impact on fatigue performance than phase transformation behavior alone (3 , 16 , 23). The interaction between these geometric factors and proprietary manufacturing processes can influence the mechanical response (24), potentially explaining the similar fatigue resistance observed between the Solla instruments, despite their differing DSC profiles. The greater reduction in cyclic fatigue resistance at body temperature observed for ProDesign Logic (26.1%) compared to Solla Colors (3.2%) and Solla Purple (8.4%) may be attributed to distinct phase distributions resulting from their proprietary manufacturing processes. While ProDesign Logic's significant fatigue reduction (26.1%) at body temperature aligns with its high As temperature (45.5°C) and predominantly martensitic structure, the more stable performance of Solla instruments (3.2-8.4% reduction) suggests they likely contain lower martensite fractions and greater austenite stability at clinical temperatures. However, the exact phase composition and distribution in these commercially available systems remain undefined. X-ray diffraction (XRD) analysis is therefore essential to conclusively determine their crystalline characteristics, including precise quantification of phase ratios (austenite/martensite/R-phase) and identification of potential microstructural heterogeneities that may influence mechanical performance (13 , 19). This study implemented a static testing protocol to assess cyclic fatigue resistance, aligning with previous studies (3 - 5 , 15 - 17 , 19). Static testing effectively eliminates torsional stress artifacts present in dynamic testing configurations that utilize bending tubes (25 , 26), while simultaneously overcoming the technical limitations associated with achieving pure axial motion in dynamic systems (27). Although alternative dynamic testing approaches are available, their heightened susceptibility to extraneous factors may mask fundamental differences in instrument performance. Consequently, static testing was chosen to ensure that the collected data accurately represents the inherent mechanical behavior of each file design, independent of the influences of the testing apparatus. Our results demonstrate that the cross-sectional design can also significantly influence cyclic fatigue resistance, with ProDesign Logic's superior performance attributable to its S-shaped geometry, which features a reduced cross-sectional area and optimized stress distribution. This finding aligns with finite element analyses, which show that instruments with smaller cross-sectional diameters exhibit lower stress concentrations during cyclic loading (28). Consistent with previous findings that S-cross-section designs enhance fatigue resistance (29 , 30), ProDesign Logic's modified S-shape showed approximately 25% greater cycles to failure than triangular/trapezoidal designs. The absence of significant differences in performance between Solla Colors and Solla Purple may be attributed to their similar core metal mass, despite their distinct geometric configurations. SEM examination revealed characteristic ductile fracture patterns across all instrument systems following cyclic fatigue testing. The fractured surfaces consistently exhibited crack initiation zones with fatigue striations, progressive crack propagation areas showing plastic deformation of lateral cutting edges, and overload zones containing numerous microvoid coalescence dimples. These observations align with failure patterns reported in previous studies of NiTi endodontic instruments (4 , 16 , 17 , 19). As the first study to evaluate both the metallurgical properties of ProDesign Logic and the mechanical characteristics of Solla Purple and Solla Colors systems, our findings provide critical benchmarks for clinical decision-making. The superior performance of ProDesign Logic recommends its use in technically demanding cases involving prolonged instrumentation of curved canals. In contrast, the Solla files exhibited less predictable fracture behavior, suggesting they may be better suited for lower-stress clinical scenarios. The results should be interpreted considering the inherent limitations of in vitro models, which cannot fully replicate the clinical environment or cumulative thermomechanical stresses (e.g., repeated sterilization cycles) (3). Future studies should expand this research by conducting clinical validation of shaping ability and torsional fatigue testing, complemented by advanced material characterization techniques such as XRD, to provide a more comprehensive analysis of these NiTi instruments.

## Conclusions

Within the limitations of this study, the metallurgical properties and instrument design significantly influence cyclic fatigue resistance. The ProDesign Logic system demonstrated superior cyclic fatigue resistance, attributable to its specific metallurgical characteristics and cross-sectional geometry. In contrast, the Solla instruments (Colors and Purple) showed comparable but lower fatigue resistance despite their distinct phase transformation profiles.

## Figures and Tables

**Table 1:** Time (seconds) and number of cycles to fracture (NCF) of the instruments.

Instruments	Time (s)		NCF	
	20°C	36°C	20°C	36°C
Logic	368.5±37.07Aa	272.5±142.5Aa	2457±247.2Aa	1817±950.0Aa
Solla Colors	170.8±50.88Ab	165.3±21.49Aa	1139±339.2Ab	1102±143.3Aa
Solla Purple	170.7±53.90Ab	156.3±9.60Aa	1138±359.3Ab	1042±64.0Aa

Values are presented as mean ± standard deviation. Different lowercase letters indicate statistically significant differences between instruments within the same temperature condition (p<0.05). Different capital letters indicate significant differences between the same instrument tested at different temperatures (room temperature vs. simulated body temperature, p<0.05).

## Data Availability

Data available on request from the authors.
